# Editorial: Host-pathogen interactions in nontuberculous mycobacterial infections

**DOI:** 10.3389/fimmu.2023.1201159

**Published:** 2023-05-26

**Authors:** Nicola Ivan Lorè, Sho Yamasaki, Rachel E. Simmonds, Eun-Kyeong Jo

**Affiliations:** ^1^ Emerging Bacterial Pathogens Unit, Division of Immunology, Transplantation and Infectious Diseases, IRCCS Ospedale San Raffaele, Milano, Italy; ^2^ Department of Molecular Immunology, RIMD/IFReC, Osaka University, Suita, Japan; ^3^ Department of Microbial Sciences, School of Biosciences, University of Surrey, Guildford, United Kingdom; ^4^ Department of Microbiology, Chungnam National University College of Medicine, Daejeon, Republic of Korea; ^5^ Infection Control Convergence Research Center, Chungnam National University College of Medicine, Daejeon, Republic of Korea

**Keywords:** nontuberculous mycobacteria, virulence factor, immunity, host-directed therapeutics, immunometabolism, lung diseases, immune cells, inflammation

Nonmycobacterial (NTM) lung infections are emerging as global health threats; however, the molecular mechanisms underlying host-microbial interactions are poorly understood compared to tuberculosis caused by *Mycobacterium tuberculosis*. NTM-host interactions are complex and dynamic processes between the mycobacterial components and host factors, influencing the infection outcomes. While NTM evolves numerous strategies to establish the infection and evade from host’s defense system, the host cells encounter the pathogenic stresses through a range of typical (immune) and non-typical components to induce defensive pathways that limit or eradicate intracellular pathogenic replications. Here, a special issue of articles discusses how NTM bacteria modulate the host defense system, in which immune and nonimmune components are functionally involved in the antimicrobial responses, and how different host cell types participate in the protective responses against NTM infections. In doing so, we outline several weapons harboring the therapeutic potential in a tug-of-war at the interface of host and NTM bacteria. A more detailed understanding of underlying NTM-host crosstalks will provide new alternative therapeutic and preventive strategies for NTM infections, which are often refractory to the conventional antibiotic-based regiments (1).

An opening field is related to the virulence factors associated with *Mycobacterium abscessus* (Mabs). So far less is known of the effector molecules in Mabs, while much more is known of the role of *M. tuberculosis*-secreted effector molecules and host response. Bar-Oz et al. briefly summarized the knowledge of secreted effectors in Mtb (such as ESX secretion, SecA2, TAT, and others), and provide known and unknown parallel pathways in Mabs infection. Overall, this represents an interesting review providing the knowledge base and framework about secretion systems in mycobacteria, phagosome permeabilization, phagosome maturation and acidification, autophagy inhibition, modulating cytokine response, and cell death manipulation.

In addition, research on the functions of immune components is crucial in the context of NTM lung diseases. In this regard, Gramegna et al. provided a macroscopic comprehensive overview of cell-mediated immunity, focusing on the functions of lymphoid cells, including natural killer cells, innate lymphoid cells, NKT cells, mucosal-associated invariant T (MAIT) cells, γδ T cells, and conventional T cells, towards the innate and adaptive immune responses against NTM infections. Importantly, the authors highlight the T cell immune exhaustion, which could be considered an adjuvant therapeutic approach based on immune checkpoint inhibitors. They also described up-to-date knowledge on the protective and pathophysiological roles of several lymphoid cells, which are belonging innate and adaptive immune responses. These lymphoid cells exhibit distinct functions, i.e., cytotoxic, immune regulatory, antimicrobial, and bridging innate and adaptive immunity during NTM lung infections. Particularly, MAIT cells can exhibit antimicrobial function, presumably mediated through cytokine generation. However, the precise mechanisms by which MAIT cell-mediated anti-NTM responses remain to be explored.

Lung airway ciliopathy is a predisposing factor for the development of progressive lung infection as demonstrated in individuals who have primary ciliary dyskinesia. The structural lung abnormality with impaired mucociliary clearance promotes the development of bronchiectasis and infection with either Mabs or *Mycobacterium avium* complex (MAC) (2). Nava et al. exploited the Cre/loxP system in mice to delete the gene IFT88, an intraflagellar transport protein necessary for the normal development of cilia. Thanks to this approach, they found that mice lacking the IFT88 gene display an impaired resistance to infection when infected with Mabs embedded in agarose beads. Moreover, Mabs infection was associated with a decreased percentage of T regulatory cells in the total lung lymphocyte population and increased levels of pro-inflammatory cytokine in the bronchial alveolar lavage fluids as observed in the IFT88 KO mice in comparison to the wild-type group. The authors concluded that ciliopathy associated with structural lung disease may play a role in NTM pulmonary infection *via* alteration of the local immunologic lung milieu.

There is growing evidence that the intracellular metabolites resulting from host cell metabolic reprogramming have significant potential for immune-modulating and protective roles during mycobacterial infection (3, 4). To elucidate the function of glycolysis and the pyruvate during *Mycobacterium avium* (Mav), Røst et al. showed that the macrophage metabolic reprogramming towards glycolysis is essential for the host defense against Mav infection through pyruvate-mediated mitochondrial reactive oxygen species (mtROS) in human monocyte-derived macrophages. More specifically, the production of pyruvate and its shuttling into the mitochondria is required for the restriction of Mav in human macrophages. The pyruvate import into the mitochondria results in the hyperpolarization of mitochondria during Mav infection. Pyruvate leads to produce mtROS through reverse electron transport (RET) *via* complex I in human macrophages. However, it remains elusive how pyruvate drives the establishment of RET and the production of mtROS during Mav infection. Future studies are warranted to clarify whether adjunctive therapy would be beneficial by using chemicals to upregulate glycolysis and mtROS production for NTM infections.

Indeed, the potential strategies for host-directed therapy (HDT) include the boosting of protective immunity and the rehabilitation of altered host responses caused by a variety of pathogens (5–7). Park et al. discuss how host susceptibility factors affect the consequences of MAC infection and which molecules/pathways participate in the enhancement of host defense against MAC infection. In addition to congenital factors, numerous acquired factors such as lung abnormalities and dysfunctional immunity are involved in the increased host susceptibility to MAC infection. Excessive or suppressed inflammatory responses are also crucial for intracellular MAC replication and pathogenesis. In this context, the regulation of the dynamic balance between destructive inflammation and host immune defense is critical for the successful management of MAC infection. Whether the potential candidates/agents for HDT contribute to better clinical outcomes should be elucidated in further experimental studies and clinical trials.

This special Research Topic mainly covers how NTM bacteria or their effectors circumvent host immune defense systems, which innate and adaptive immune components exert to control or promote chronic NTM infection in a context-dependent manner. Identifying both-side factors, i.e., bacterial virulence and host susceptibility is fundamental to understanding the intricate mechanisms for the dialogues between NTM and host. Despite the recent advances, there is an urgent need for further study to elucidate the molecular events underlying NTM-host interaction and to utilize the host immune arms for developing therapy approaches.

## Author contributions

All authors reviewed and edited the manuscript. E-KJ conceptualized and supervised the manuscript. NL and E-KJ wrote the manuscript. NL, SY, RS, and E-KJ reviewed and edited the manuscript. This manuscript was peer-reviewed before submission. All authors contributed to the article and approved the submitted version.
